# Review of possible mechanisms of radiotherapy resistance in cervical cancer

**DOI:** 10.3389/fonc.2023.1164985

**Published:** 2023-08-22

**Authors:** Hanqun Zhang, Xiaohu Wang, Yan Ma, Qiuning Zhang, Ruifeng Liu, Hongtao Luo, Zi Wang

**Affiliations:** ^1^ The First School of Clinical Medicine, Lanzhou University, Lanzhou, China; ^2^ Department of Oncology, Guizhou Provincial People's Hospital, Guizhou, China; ^3^ University of Chinese Academy of Sciences, Beijing, China; ^4^ Institute of Modern Physics, Chinese Academy of Sciences, Lanzhou, China; ^5^ Lanzhou Heavy Ion Hospital, Lanzhou, China

**Keywords:** cervical cancer, radiotherapy, radiotherapy resistance, mechanism, radiotherapy sensitivity

## Abstract

Radiotherapy is one of the main treatments for cervical cancer. Early cervical cancer is usually considered postoperative radiotherapy alone. Radiotherapy combined with cisplatin is the standard treatment for locally advanced cervical cancer (LACC), but sometimes the disease will relapse within a short time after the end of treatment. Tumor recurrence is usually related to the inherent radiation resistance of the tumor, mainly involving cell proliferation, apoptosis, DNA repair, tumor microenvironment, tumor metabolism, and stem cells. In the past few decades, the mechanism of radiotherapy resistance of cervical cancer has been extensively studied, but due to its complex process, the specific mechanism of radiotherapy resistance of cervical cancer is still not fully understood. In this review, we discuss the current status of radiotherapy resistance in cervical cancer and the possible mechanisms of radiotherapy resistance, and provide favorable therapeutic targets for improving radiotherapy sensitivity. In conclusion, this article describes the importance of understanding the pathway and target of radioresistance for cervical cancer to promote the development of effective radiotherapy sensitizers.

## 1 Background

Cervical cancer (CC) is the most common cancer and cause of cancer death in women, with an estimated more than 600,000 new cases and more than 300,000 deaths globally in 2020. Therefore, CC is still a major public health problem threatening women’s lives and health (1). A common cause of CC is human papillovirus (HPV) infection (2). Other risk factors for CC include sexually transmitted infections (STIs), smoking, an increasing number of births, and long-term use of contraceptives (3). CC mainly occurs in developing countries. Owing to hidden incidence and poor medical and health conditions, more than two-thirds of patients have advanced to a locally advanced stage at the time of treatment [International Federation of Obstetricians and Gynaecologists, stage IB2-IVA]. Concurrent chemoradiotherapy is the standard treatment regimen for LACC. However, only 30%–80% of patients with CC survive to 5 years, and the treatment prognosis of CC is still not satisfactory (4, 5).

## 2 Relevant definitions and possible mechanisms of radiation therapy resistance

The free radicals produced by radiotherapy may directly or indirectly affect DNA and other cellular molecules. These free radicals induce the formation of reactive oxygen species (ROS) and oxidative stress, leading to tumor cell death (6). However, radiation therapy can also cause cancer cells to develop radiation resistance to ionizing radiation (7), leading to cancer treatment failure. Radiotherapy resistance, defined as cancer cell resistance to radiotherapy rays, results in the failure of antitumor therapy and tumor recurrence or metastasis (8), and is a major obstacle to cancer treatment. When the gene or phenotype of cancer cells is changed due to radiotherapy, radiotherapy resistance is called intrinsic radiotherapy resistance, while radiotherapy resistance is called acquired radiotherapy resistance due to the protection of the cancer microenvironment (9). Radiation resistance leads to poor treatment outcomes, cancer recurrence, poor patient prognosis, decreased quality of life, and increased financial burden of disease treatment. In addition, radiation resistance also leads to damage to normal tissues around the tumor; damages the function of normal tissues; causes radiation-related inflammation, bleeding, and other symptoms (10–12); and may lead to secondary malignant tumors (13).

Radiotherapy is one of the main cancer treatment methods; approximately 60% of patients with malignant tumors need radiation therapy (14), and when radiotherapy is combined with other therapies (surgery, chemotherapy, and immunotherapy), it can improve the surgical resection rate, local control rate, and survival rate of patients with malignant tumors (15). Despite the improvement of radiation therapy techniques and methods, cancer recurrence or metastasis is eventually caused by the inherent or acquired radiation resistance of cancer cells (16, 17). At present, the mechanism of radiotherapy resistance in malignant tumors is not very clear, and the possible mechanism is as follows: (1) Tumor hypoxia: significantly increased HIF1-α activity induces radiation resistance in the case of hypoxia (18). In addition, under hypoxic conditions, radiation therapy can lead to free radical production, which leads to reduced oxidative stress, and also leads to radiation resistance (19). (2) Tumor microenvironment: the tumor microenvironment includes tumor cells, cell matrix, tumor blood vessels, and immune cells. Radiation therapy can cause vascular damage and induce the production of cytokines/chemokines recruited by immune cells, leading to tumor hypoxia and triggering an immune response (20, 21).. The increase in immunosuppressive cells in the tumor microenvironment after radiotherapy leads to radiotherapy resistance (22–24). (3) DNA damage repair: Radiotherapy can cause DNA double- or single-strand breaks, as well as chromosome variation, apoptosis, or cell death (25). The ataxic telangiectasia mutation (ATM) pathway (26), nonhomologous terminal junction (27), single-strand break repair (28), and homologous recombination pathway cooperate to promote the repair of DNA damage (29) and other repair genes in tumor, which is also one of the main reasons leading to radiation resistance. (4) Tumor stem cells: the main characteristics of tumor stem cells are self-renewal, differentiation to other tumor cells, and the ability to resist tumor therapy, which is one of the causes of local tumor recurrence and distant metastasis. The increase in tumor stem cells and the radiation resistance of tumor stem cells themselves can lead to radiotherapy resistance (30). In addition, some nontumor stem cells may develop into tumor stem cells after radiotherapy (31). (5) Tumor metabolism: In malignant tumors, tumor cells are significantly correlated with metabolic disorders (32), and metabolic reprogramming is also considered to be one of the important manifestations of malignant tumors (33). However, the disorder of tumor cell metabolism is related to radiotherapy resistance (34). For example, glucose metabolism and regulation of mitochondrial function can both affect radiotherapy sensitivity (35, 36). (6) Cell cycle, apoptosis, and other signaling pathways: HR (homologous recombination) and NHEJ (nonhomologous end-joining) are identified early in the history of double-strand break (DSB) repair, and HR pathways are activated by late S and G2/M phases of cells (37). However, NHEJ is activated in G0/G1 and G2/M phases (38), and both the HR pathway and NHEJ pathway are associated with radiotherapy sensitivity in malignant tumors (39). Radiation therapy can cause DNA damage in tumor cells and lead to apoptosis of tumor cells, and tumor cells escaping from apoptosis may be one of the main causes of radiation resistance in tumor cells (40). On the other hand, the expression of apoptotic proteins is involved in the generation of radiation resistance (41). (7) Others: In addition, the mechanism of radiotherapy resistance of tumor cells is also closely related to tumor heterogeneity (19), microRNAs (42), and long noncoding RNA (lncRNAs) (43).

## 3 Possible mechanism of resistance to radiotherapy for cervical cancer

**Figure 1:**
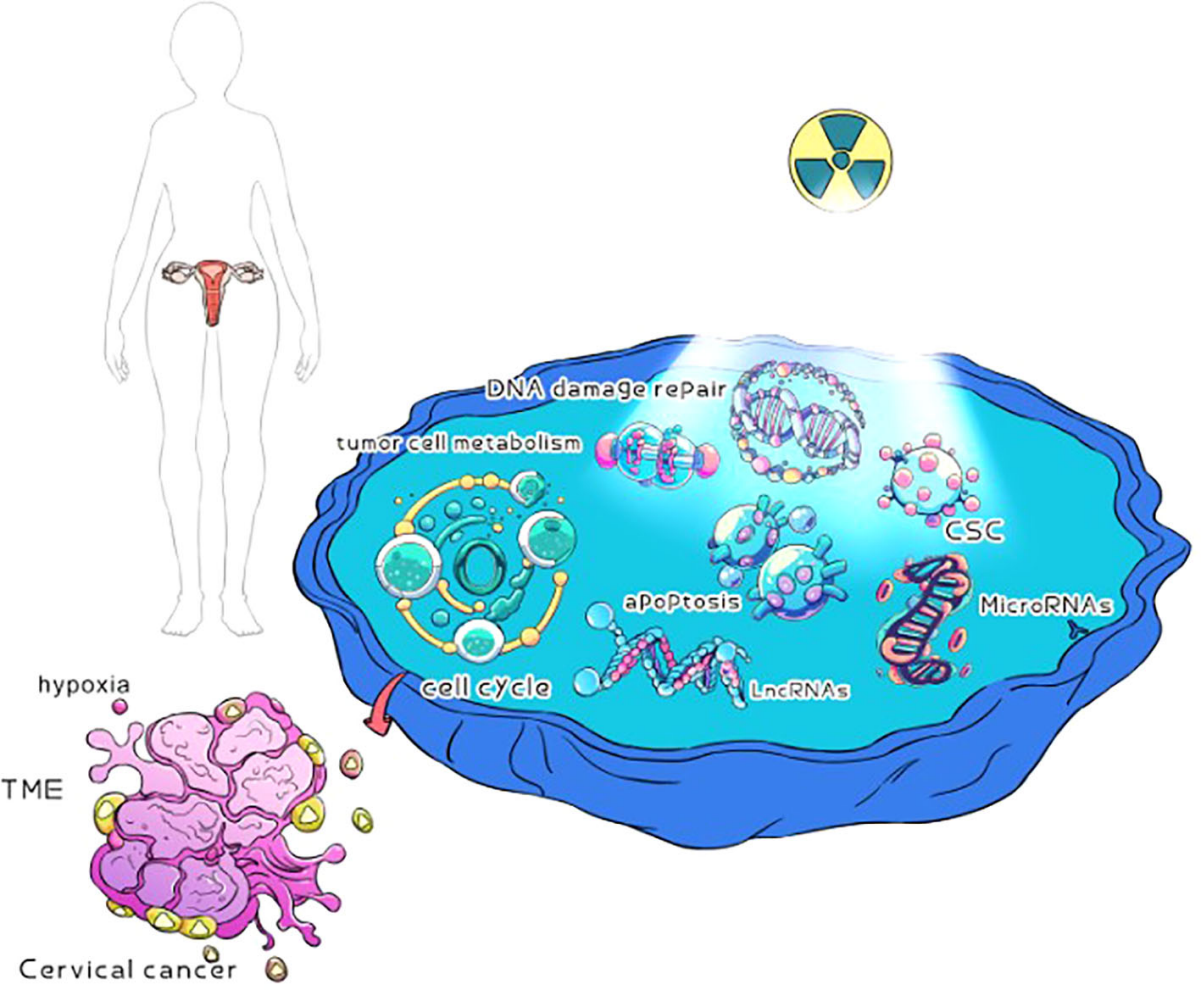
Possible mechanisms of radiotherapy for cervical cancer. Cervical cancer cells may be hypoxia, DNA damage repair, TME, tumor cell metabolism, CSC, cell cycle, apoptosis, microRNAs, and lncRNAs in radiotherapy resistance.

CC is an important cause of female death (44). Radiation therapy plays an important role in the treatment of CC, and is used for any stage of CC (45). External pelvic irradiation combined with brachytherapy is the standard treatment for CC (46). Single- and double-strand DNA breakage induced by radiotherapy is one of the main mechanisms of killing tumor cells. One is to damage the DNA of tumor cells directly by radiation, so as to kill tumor cells; The other is that radiation leads to the decomposition of water and the formation of free radicals, which leads to the indirect death of tumor cells (47). Throughout the diagnosis and treatment of CC, approximately 80% of patients with CC need to receive radiation therapy (48). However, at the end of each radiotherapy, when the damaged tumor cells avoid tumor cell death through alternative mechanisms to facilitate their repair, proliferation, and escape, the tumor cells will develop resistance to radiotherapy (49). In addition, in the microenvironment of CC tissue, local tumors may have hypoxia and tumor stem cells (19, 30), which may also lead to the resistance of CC cells to radiotherapy. Therefore, radiotherapy resistance is still the main reason for the low overall survival rate and disease-free survival rate of patients with CC (50), as well as one of the main reasons for local recurrence and distant metastasis of CC (51). Radiotherapy resistance is a complex and mysterious biological process, the mechanism of which is still unclear. Over the past few decades, many basic and clinical studies have been conducted to eliminate the effects of radiation therapy resistance on CC outcomes. The potential mechanism of radiotherapy resistance to CC has been analyzed through the relationship between radiotherapy resistance to CC and the hypoxia of tumor cells, DNA damage repair, tumor microenvironment, and tumor stem cells. It provides a therapeutic target for sensitizing CC to radiotherapy (**Figure 1**).

### 3.1 Resistance to radiotherapy of cervical cancer and hypoxia of tumor cells

Hypoxia of tumor cells is very common in malignant tumors, and is characterized by hypoxia of tumor cells (52). CC in a hypoxic state is associated with tumor metastasis, extracellular matrix (ECM) remodeling, and patient prognosis (53, 54), and CC tends to cause hypoxia and is an independent risk factor (55). Hypoxia can cause resistance to treatment, especially radiotherapy. Hypoxia can produce ROS, alter the expression of proteins associated with double-stranded DNA repair, and then lead to the disturbance of cell cycle checkpoint control, resulting in abnormal DNA repair pathways (56–58), which can lead to tumor recurrence or metastasis after treatment. Hypoxic inducible factor (HIF) is a transcriptional activator associated with the adaptive response of cells to hypoxia. To date, three members of the HIF family have been identified (HIF-1α, HIF-2α, and HIF-3α) (59). It has been found that HIF-1α, HIF-2α, and HIF-3α are related to radiotherapy resistance in CC, and HIF-1α has been extensively studied. HIF-1 is one of the main factors regulating the hypoxia signaling pathway. HIF-1 is a heterodimeric transcription factor consisting of HIF-1α and HIF-1β subunits. HIF-1α can be rapidly degraded under normal oxygen conditions but is stable under low-oxygen conditions. Thus, hypoxia can lead to HIF-1 expression, and its increased expression can lead to the expression of multiple gene products involved in a variety of biological processes (60). Fu et al. irradiated cervical HeLa cells with 8 Gy under hypoxic conditions, and found that the expression of VEGF and HIF-1α was enhanced in HeLa cells under hypoxic conditions after irradiation, while the expression of P53 was inhibited, resulting in radiation resistance. The expression of VEGF and HIF-1α was declined after transfection with HIF-1α siRNA irradiation. However, the increased expression of P53 increases the radiation sensitivity. The results indicate that HIF-1α is one of the key genes in the radiation resistance pathway mediated by increasing the expression of VEGF and inhibiting the expression of P53 under hypoxia (61). Similar studies have also confirmed that hypoxia stimulates the expression of VEGF and HIF-1α in HeLa cells and induces radiation resistance. Overexpression of VEGF promotes radiation resistance, while unactivated HIF-1α or blocking VEGF–VEGFR interactions eliminates hypoxia-induced radiation resistance (62). The HIF-1α positive expression rate was 45% (17/38) in CC tissue, and the 10-year recurrence-free survival rate (RFS) was approximately 22% in patients with HIF-1α-positive tumors and 68.7% in patients with HIF-1α-negative tumors after external and intracavitary irradiation (*p* = 0.04). HIF-1α can be used as a prognostic indicator of radiotherapy for CC (63). HIF-2α and HIF-1α have distinct effects (64–66), but they are the same in causing radiotherapy resistance, and this common effect has been demonstrated in head and neck tumors (67). In addition, Kim et al.’s immunohistochemical study of HIF-2α expression in 36 cases of cervical squamous cell carcinoma (CSCC) (24 radiation-sensitive and 12 radiation-resistant) showed that the fraction of HIF-2α expression in the radiation-sensitive group was lower than that in the radiation-resistant group, and that HIF-2α expression was positively correlated with radiotherapy failure or local recurrence. Furthermore, the prognosis of patients with CC is related to the association between stained area and risk of recurrence (68). There are few studies on HIF-3α, and it has been reported that increased HIF-3α expression is associated with pancreatic cancer metastasis and ovarian cancer progression (69). Reanalysis of CC using the TCGA database showed expression patterns between normal and tumor samples, suggesting that high expression of HIF-1α and HIF-3α led to decreased survival in patients with CC. The increased expression of HIF-3α could promote the expression of miR-603, and the high expression of miR-630 could lead to the apoptosis of HeLa cells induced by radiotherapy, while the silencing of miR-630 could accelerate the apoptosis induced by radiotherapy. These results indicate that miR-630 has an anti-radiation function, and the high expression of miR-630 can also promote the expression of HIF-3α, and both have a positive regulatory ring effect (70).

Therefore, the oxygen content of cells in CC tissue not only has an important impact on the treatment, but also has a very important significance for the prognosis of patients. How to increase oxygen content or block the occurrence of hypoxia and improve the sensitivity of CC radiotherapy will be an important topic for the treatment and prognosis of patients with CC. HIF is one of the important factors that hinder the sensitivity of CC radiotherapy. Targeting HIF or blocking its action pathway may become one of the important methods to improve the sensitivity of CC radiotherapy.

### 3.2 Resistance to radiotherapy in cervical cancer and DNA damage repair

The most lethal radiotherapy-induced DNA damage is DNA DSBs, which can cause a range of cellular DNA damage responses (DDRs), including responses that help tumor cells repair from radiation therapy damage, such as cell cycle arrest, DNA damage and transduction pathway activation, and DNA repair. Apparently, these protective DDRs confer resistance to radiation therapy on tumor cells (9). HR and NHEJ are the main pathways for the repair of DNA DSBs induced by radiotherapy in tumor cells. Therefore, the upregulation of DNA repair pathways is considered to be the main acquired mechanism by which cancer cells may become resistant to radiotherapy (71). At the center of DDR is primarily ataxic telangiectasia mutant (ATM) kinase. In addition, ATM plays an important role in DNA DSBs, DNA repair, apoptosis, and the cell cycle. When DNA DSB is induced, ATM is activated by autophosphorylation of Ser1981 (Ser1981), followed by phosphorylation of proteins involved in apoptosis and DNA repair factors (72–75). Activation of DNA repair genes for DSBs is one of the causes of radiation therapy resistance (76). Radiation therapy in advanced CC mainly causes cell death by inducing DNA damage, while tumor cells induce DNA repair by activating the DDR, which leads to radiotherapy resistance. Roossinck et al. used immunohistochemical methods to detect p-ATM in tissues of 349 patients with CC. Among them, p-ATM was positive in 344 patients, indicating that ATMs were activated at least to some extent in most patients, and p-ATM was highly expressed in 183 patients. High expression of p-ATM can lead to decreased survival of patients with CC. Further *in vitro* experimental studies have shown that high expression of ATM can increase the radiotherapy resistance of CC cells, while downregulation of ATM expression can lead to increased radiotherapy sensitivity of CC cells (77). At present, the direct regulation of p53 by ATM has been confirmed (73), and p53 is mainly related to DNA damage repair, the cell cycle, and apoptosis (78, 79). However, high expression of p53 may lead to resistance to radiotherapy. Almeida et al. detected tissues and CC cell lines (Caski and C33A) of 10 patients with CC before and after radiotherapy and found that the expression of p53 in CC tissues after radiotherapy was higher than that before radiotherapy, but no high expression of p53 was found in the *in vitro* experiment. Different results may exist in the model of *in vitro* and *in vivo* studies (80). In addition, a retrospective analysis of 78 cases of contemporaneous CC (II A and B) found that doses ranged from 35 Gy to 50 Gy at 200 cGy each time. Surgery was performed 4–6 weeks after the end of radiotherapy. In 78 tissue samples examined after surgery, 51% (40 cases) responded completely to treatment with no tumor cells. Forty-nine percent (38 cases) had residual tumors that were considered resistant to radiation therapy. p53 was detected in 38 patients with radiotherapy resistance, and it was found that 34% (13 cases) of patients had increased expression of p53 in CC tissues, while no p53 expression was found in the radiotherapy-sensitive group, so it was considered that p53 might be one of the causes of radiotherapy resistance to CC (81). Currently, it has been reported that activation of the ATM/p53 pathway can enhance DNA damage repair and thus increase radiation therapy resistance (82). However, the ATM/p-53 pathway may be one of the mechanisms that increase DNA damage repair and lead to decreased sensitivity to radiotherapy in CC resistance.

HR and NHEJ are the two most common DNA damage repair mechanisms following radiotherapy (76, 83, 84). In addition, the downstream factors of HR and NHEJ are potential therapeutic targets for regulating radiotherapy sensitivity (85). RAD51 is a conserved protein that activates DNA repair via the HR pathway and regulates cell sensitivity to radiation therapy (86). The decreased expression of RAD51 can inhibit DNA damage repair and enhance the radiotherapy sensitivity of CC cells, while the increased expression of RAD51 can enhance DNA damage repair, thus leading to the radiotherapy resistance of CC cells (87, 88). DNA-PKCs, Ku70, Ku80, and Ku86 proteins were found to be involved in DNA DSB repair mainly through the NEHJ pathway and lead to radiotherapy resistance in CC (89–91). In addition, MRN and PARP1 are also involved in DSB damage repair through the NHEJ pathway, leading to radiotherapy resistance in CC (92, 93).

Radiation therapy induces DNA DSBs, which cause apoptosis, cell cycle checkpoint changes, and partial pathway changes in cancer cells, leading to the death of cancer cells. This is the normal death process of cancer cells after radiation therapy. However, after radiotherapy, cancer cells in the body can evolve an individual DNA damage repair mechanism through their inherent radiotherapy resistance mechanism or acquired radiotherapy resistance mechanism, so as to escape cell death and lead to the failure of radiotherapy. How to overcome or inhibit DNA repair after damage is an important task and challenge to improve the resistance to radiotherapy for CC. In addition, the ATM/p-53 and HR/NHEJ pathways are important pathways leading to radiotherapy resistance, and inhibition or blocking of these pathways may be an important means to improve the radiosensitivity of CC.

### 3.3 Radiotherapy resistance to cervical cancer and the tumor microenvironment

The tumor microenvironment is composed of the ECM, immune cells (including lymphocytes and NK cells), and stromal cells (such as mesenchymal stromal cells, adipocytes, vasculature, and fibroblasts) (94). In solid tumors, with the continuous proliferation of tumor cells, the tumor volume gradually increases, and corresponding changes will occur in the TME (95). In advanced solid tumors, TMEs are highly complex and highly heterogeneous. In addition, exosomes play an important role between TME cells and are also involved in the transformation of non-tumor cells adjacent to the TME into tumor cells. The ECM in the TME is usually dense and rigid, leading to adhesion formation. Furthermore, the rapid growth of tumor cells leads to a lack of hypoxic areas and nutrients in the tumor, resulting in the Warburg effect. In the TME, the rapid growth of tumor cells induces tumor angiogenesis, which leads to the formation of a chaotic branching structure. In addition, lymphocytes, cancer-related fibroblast myeloid cells, and mesenchymal cells in TME play an important role in the development and prognosis of malignant tumors (96–98). The TME is considered to be an important determinant of tumor biological behavior and treatment response in patients with CC (99–101). Current studies have shown that the TME is closely related to radiotherapy resistance in CC (85). In the TME, there is an imbalance between tumor cell growth and neovascularization. Abnormal functional and morphological tumor vessels can lead to a hypoxic state in the TME (102), and hypoxia can lead to radiotherapy resistance in CC (60–62). In addition, the TME can affect tumor metabolism, and the abnormal tumor metabolism helps promote the resistance to radiation therapy in CC, which will be elaborated upon in the following chapters. G-MDSCs (granulocytic-myeloid derived suppressor cells) are immature myeloid cells with different functional properties from normal mature neutrophils. CC is associated with a high concentration of circulating granulocyte MDSCs (G-MDSCs), including prominent immunosuppressive and angiogenic activities (103). However, G-MDSCs can cause radiotherapy resistance and produce a large amount of the angiogenic factor Bv8 (104), leading to tumor angiogenesis and TME hypoxia (102), which, in turn, can cause radiotherapy resistance in CC (60–62). In addition, the expression of PD-1 and PD-L1 is also associated with radiotherapy resistance in CC (105, 106). Radiotherapy plays an important role in CC and also causes radiotherapy resistance by changing the factors in the TME. Radiotherapy can induce EMT to promote metastasis and enhance the invasion of cancer (107). The transcription factors induced by ionizing radiation can activate a variety of EMT processes, including Snai1, Snai2, HIF-1, ZEB1, STAT3, and Twist (108–114). These transcription factors activate the corresponding signaling pathway and enhance the capacity of tumor cells to undergo EMT, which can cause resistance to radiation therapy. In addition, EMT-induced transcription factors confer cancer stem cell (CSC) characteristics on cells and promote CSC production. CSCs are considered to be radiation-resistant cells that function primarily by reducing DNA damage caused by radiation therapy and enhancing DNA repair (115).

The TME plays an important role in radiotherapy resistance in CC. The existence of immune cells and hypoxia in the TME is closely related to radiation resistance in CC. In addition, glycolysis and lipid metabolism in the TME are also important causes of radiotherapy resistance in CC. How to target the components or pathways in the TME to reduce the resistance to radiotherapy for CC is one of the important ways to improve the curative effect of radiotherapy for CC. However, the exact mechanism between the TME and radiotherapy resistance is still unclear. TMEs contain immune factors, hypoxia-inducing factors, tumor vessels, and inflammatory factors. They also participate in lipid metabolism, glycolysis, and amino acid metabolism. These factors and metabolism may act on radiotherapy resistance separately or jointly. We believe that with the continuous development of research, the mechanism of radiotherapy resistance induced by the TME will be gradually clarified in the future. Increase the sensitivity of CC to radiotherapy by targeting related factors or blocking metabolic pathways.

### 3.4 Radiotherapy resistance of cervical cancer and tumor cell metabolism

Among human solid tumors, approximately 60% of malignant tumors mainly use glycolysis for energy generation and self-survival (116). Glycolytic metabolism was first proposed by Warburg. Even in the presence of high oxygen, malignant tumor cells have a high glycolytic rate (117). In a low-oxygen environment, the decreased adaptability of mitochondrial respiration also leads to hyperglycolysis, a phenomenon known as the “Pasteur effect” (118). A high glycolysis rate leads to increased tumor lactate concentration, which is associated with poor clinical prognosis of CC patients after radiotherapy and with radiotherapy resistance (119, 120). The metabolic disorders of malignant tumor cells, including lipid metabolism, glucose metabolism, and amino acid metabolism, may help regulate the radiation resistance of CC (121–123). Glucose transporter 1 (GLUT1) is one of the most common and widely distributed glucose transporter protein families. GLUT1 is a family of glucose transporter proteins that encases the cell membrane (124). In CC, high expression of GLUT1 is more likely to lead to radiotherapy resistance, and GLUT1 can be used as one of a biomarker of radiotherapy resistance in CC (48, 125). The possible mechanism is that after glucose enters tumor cells by GLUT1, it rapidly undergoes glycolysis and produces excessive lactic acid, which enhances the radiation resistance of CC. In addition, glucose in tumor cells may produce glutathione GSH via the pentose phosphate pathway (PPP). The antioxidant GSH weakens DSBs after RT, leading to radiation resistance in CC cells (85). LDHA is an NAD-dependent kinase of homologous or heterotetramer molecules located primarily in the cytoplasm. LDHA plays an important role in the process of glycolysis, converting pyruvate into lactate and NADH into NAD (126). Studies have shown that compared with the chemoradiotherapy-sensitive group, the expression of LDHA in tumor tissues of the chemoradiotherapy-resistant group is upregulated (127), and LDHA is also relatively highly expressed in CC cells. Such a high expression level is resistant to radiotherapy, and the possible mechanism is that high expression of LDHA helps cancer cells perform aerobic glycolysis and promote radiation resistance (128). Pyruvate kinase (PK) type is a key enzyme in glycolysis and its M2 subtype (PKM2) is upregulated in many malignant tumors (129). In CC, high expression of PKM2 is associated with poor prognosis in patients with CC and contributes to the generation of radiation therapy resistance (130). It has been reported that knockout of PKM2 in SiHa and HeLa cells can promote DNA DSBs and lead to G2/M cell cycle arrest, thus enhancing the radiotherapy sensitivity of CC cells (131). In addition, hexokinase 2 (HK2) also enhances CC radiation therapy resistance by participating in glycolysis (132, 133).

It has been reported that lipid metabolism is related to sensitivity to radiotherapy for CC (134). Cyclooxygenase (COX) is the enzyme responsible for converting arachidonic acid into prostaglandins. COX currently has two isomers, COX-1 and COX-2. COX-1 is constitutively expressed in a variety of cell types, and COX-2 is an inducible enzyme. There is increasing evidence that COX-2 expression is upregulated in human cancers and is associated with tumor invasion and metastasis (135, 136). Anoopkumar-Dukie et al. selectively increased the radiation sensitivity of anoxic HeLa cells by using the COX-2 inhibitor NS398, while the COX-1 inhibitor SC560 did not affect the radiation sensitivity. This indicates that both the expression and activity of COX2 contribute to the radioresistance of CC cells. Further mechanistic studies suggest that COX-2 may increase cervical radiotherapy resistance by regulating P53 phosphorylation (123). Amino acids are important substances in the biosynthesis of malignant tumor cells, and amino acid metabolism is related to the occurrence and development of malignant tumor (137). Glutamine plays an important role in the biosynthesis process by providing nitrogen and carbon (138, 139). It has been reported that there is a correlation between glutamine metabolism and radiosensitivity of malignant tumors (140). Xiang et al. studied 15 adjacent normal cervical specimens and 144 human CC specimens (86 radiation-sensitive and 58 radiation-resistant specimens) and found that the expression of phosphate-activated mitochondrial glutaminase-2 GLS2 in the radiation-sensitive CC group was lower than that in the radiation-resistant group. *In vitro* studies showed that GLS2 expression in the radiation-resistant cell line HeLaR was significantly higher than that in its parent cell line HeLa, while the GLS2 silenced cell line HeLaR showed significantly enhanced radiation sensitivity. *In vivo* studies also showed the same results; GLS2 silenced HeLaR xenografts were more sensitive to radiation. Molecular mechanism studies have shown that GLS2 regulates intracellular ROS levels by regulating the production of antioxidants GSH, NADH, and NADPH, leading to radioresistance of HeLa cells (141).

The metabolic disorder of tumor cells is not only related to tumor development, but also closely related to tumor prognosis. In addition, glucose metabolism, amino acid metabolism, and lipid metabolism may contribute to the regulation of CC radiation resistance. There is no doubt that targeting key enzymes and metabolic intermediates is a promising strategy that will potentially improve radiosensitivity in patients with CC. With the development of modern omics technology, in-depth research on the key mechanisms of metabolism has been carried out to clarify the different metabolic states of tumor cells in patients with CC, which may provide a basis for personalized treatment of patients with CC, thus improving the current treatment status of patients with resistance to CC radiotherapy.

### 3.5 Resistance to radiation therapy for cervical cancer and cancer stem cells

Radiotherapy is one of the most effective forms of treatment for malignant tumors. However, radioresistance can lead to tumor recurrence and even lead to patient death. CSCs have better DNA repair capabilities, and CSCs have been shown to lead to radiotherapy therapy resistance (142). Markers of CSCs include sry-related HMG box (SOX2) and octamer binding transcription Factor 4 (OCT4). They are believed to be related to the regulation of apoptotic pathways, telomerase function, and DNA damage repair (143–145), and the radioresistance of CC (146). Rachmadi et al. conducted immunohistochemical analysis on the expression of SOX2 and OCT4 in the tissues of 48 patients with cervical squamous cell carcinoma, and found that the expression levels of SOX2 and OCT4 were high in the tissues of patients with partial remission after radiotherapy and chemotherapy, but low in the tumor tissues of patients with complete remission after radiotherapy and chemotherapy. High expression levels of SOX2 and OCT4 are related to radioresistance in CSCC (146). A similar study in China of 132 cases of locally advanced cervical squamous cell carcinoma (LACSCC) showed that, according to progression-free survival (PFS), 132 patients were divided into a radiotherapy-resistant group and a radiotherapy-sensitive group. The expression levels of SOX2 and OCT4 were detected by immunohistochemistry. The overexpression ratio of SOX2 and OCT4 in the radiotherapy-sensitive group was significantly lower than that in the radiotherapy-resistant group, and the expression status of SOX2 and OCT4 was correlated with PFS in patients with CC. This study demonstrates that the stem cell biomarkers SOX2 and OCT4 proteins can be used to predict radiotherapy resistance in patients with LACSCC. Meanwhile, studies have also demonstrated that the expression levels of SOX2 and OCT4 are important predictors of poor survival and prognosis in patients with LACSCC (147). In addition, P16^INK4A^ is a biomarker of CC occurrence and reduction of stem cell proliferation. Studies have shown that the absence of P16^INK4A^ promotes the chemotherapy resistance and radioresistance of CC cells, and increases the expression of SOX2 (148), and the increased expression of SOX2 is associated with the radiotherapy resistance of CC (146, 147). In 2003, CD44 and CD24 were identified as stem cell surface markers in breast cancer, acting as adhesion molecules (149). Abdel-Hamid et al. (150) used CD44 and CD24 antibodies to classify HeLa cells, and the classified subpopulations were as follows: CD44−: 18.55%,CD44+CD24+: 28.17%, and CD44+CD24−: 53.28%. When cells were exposed to 5 Gy x-ray, the growth ability of the CD44+CD24+ cell subpopulation after irradiation was superior to that of other subgroups, considering that CD44+CD24+ cells had stem cell properties and radiotherapy resistance properties.

At present, treatment failure of locally advanced CC may be related to CSC, but there is no clear detection means or treatment strategy for CSC. Since local tumors may be related to the control of tumor stem cells, radiobiological and radiophysical studies should focus on the effects of radiation on tumor stem cells. CSC therapeutic targets or drug targeting vectors should be actively sought. Targeting such CSCs with different chemotherapies, biologic drugs, nanometals, or a combination of these approaches breaks new ground for improving the sensitivity of CC radiotherapy.

### 3.6 Resistance to radiation therapy for cervical cancer and cell cycle or cell apoptosis

Radiation causes DNA damage, particularly DNA DSBs, which trigger cell cycle arrest and provide time for DSB repair. When cells are irradiated, DNA damage occurs in the G1 phase, after which the G1/S checkpoint is activated, and G1/S checkpoint can be activated through at least two signaling pathways, namely, ATM/p53/p21 and ATM/CHK2/CDC25C (151). ATM was activated after irradiation, and it phosphorylated p53, promoted the dissociation of p53, and inhibited the transfer of p53 from the nucleus to the cytoplasm. On the other hand, CHK2 is activated to phosphorylate and stabilize p53, and the increase in p53 levels triggers downstream transcription of the p21 gene, leading to G1/S block (152–154). In the ATM/CHK2/CDC25C pathway, after DNA damage is induced by irradiation, CDC25C is degraded by ATM/CHK2 and G1/S arrest can be induced (155, 156). Through G1/S phase cell arrest, DNA damage repair is facilitated, leading to radiotherapy resistance. Studies have shown that specific protein 1 (Sp1) is highly expressed in CC tissues, the expression of Sp1 increases in a dose-dependent manner at the mRNA and protein levels in CC cell lines after radiation irradiation, and overexpression of Sp1 can significantly reduce the G2/M block of CC cells, leading to radiation resistance. Further mechanistic studies suggest that Sp1 may inhibit G2/M block by acting on CDK1, thereby promoting radiotherapy resistance (157). In radiation-resistant CC cell lines (HelA-R and Siha-R) and their corresponding parent cell lines (HeLa and SiHa), lncRNA NEAT1 expression was higher in radiation-resistant CC cell lines than in their corresponding parent cell lines. Considering that the high expression of lncRNA NEAT1 might be related to the radiation resistance of CC cell lines, however, downregulation of NEAT1 expression of lncRNA induced increased cell number at the G0/G1 phase, decreased cell number at the S phase, and decreased CDK2 protein levels, while cleaved caspase-3 and caspase-9 protein levels were significantly upregulated. These results demonstrate that lncRNA NEAT1 enhances the radiation resistance of CC cells by affecting the cell cycle and apoptosis (158).

The cell cycle and apoptosis are closely related to the survival of tumor cells and also affect the sensitivity of tumor to radiotherapy. By blocking the repair of tumor cells and promoting apoptosis to increase the sensitivity of tumor cells to radiation therapy, it is possible to contribute to increasing the sensitivity of CC to radiation therapy by acting on related pathways.

### 3.7 Resistance to radiotherapy for cervical cancer and microRNAs

MicroRNAs (also known as miRNAs) are single-stranded RNAs composed of approximately 21–23 nucleotides. MicroRNAs are widely found in animals, plants, and single-celled eukaryotes (159, 160). Because their bases complement the 3' untranslated region (3' UTR) of messenger RNA (mRNA), they can regulate target genes after transcription, including the entire cell signaling pathway (161, 162). Many studies have shown that abnormal miRNA expression is related to drug resistance, recurrence, and metastasis, and the prognosis of patients with malignant tumors (163–166). In addition, abnormal miRNA expression is related to radiotherapy resistance in patients with malignant tumors (167–169). Concurrent chemotherapeutic therapy is the standard treatment for LACC or recurrent cancer (170, 171). Currently, miRNAs have been found to play an important role in promoting the sensitivity of CC cells to chemotherapeutic therapy.

Given that the low expression of miR-4778-3p may be linked to the resistance of CC to radiotherapy, tissue analysis of patients with recurrent or metastatic CC revealed that the expression of miR-4778-3p was dramatically lowered. Further *in vitro* research revealed that the miR-4778-3p gene was upregulated in the radiation-resistant CC cell lines HeLa and SiHa. The results revealed that the upregulated miR-4778-3p cells significantly reduced the proportion of G2/M phase cells and significantly increased apoptosis. Moreover, miR-4778-3p upregulation resulted in an increase in the expression of markers linked to apoptosis. Moreover, miR-4778-3p selectively binds NR2C2 and Med19 and inhibits the production of both genes. Hence, targeting and controlling the expression of NR2C2 and Med19 may increase the susceptibility of CC to radiotherapy while decreasing miR-4778-3p expression may increase the resilience of CC cells to radiotherapy (172). Another study reaffirmed the link between low miRNA expression and CC radiation resistance. By qRT-PCR analysis, the expression level of miR-449b-5p in CC tissues and cell lines was relatively lower than that in normal tissues and cells. Moreover, downregulation of miR-449b-5p is closely related to the low overall survival rate of CC patients. *In vitro* studies showed that upregulation of miR-449b-5p inhibited cell proliferation, migration, and invasion. In addition, FOXP1 was found to be a downstream target of miR-449b-5p. The experimental verification showed that miR-449b-5p-FOXP1 had the biological function of a feedback loop. In summary, miR-449b-5p regulates cell proliferation, migration, and radiation resistance in CC by interacting with the transcriptional suppressor FOXP1 (173). In addition, miR-15a-3p, miR-132, miR-499a-5p, miR-320, miR-29a, and miR-4429 were all expressed at low levels in CC tissues or CC cell lines. This low expression level is associated with the resistance to radiotherapy of CC (87, 174–178). However, in the miRNA world, some miRNAs are still highly expressed in cancer tissues and cells, which is also related to drug resistance, radiation resistance, recurrence, and metastasis of tumor patients (179, 180). The results showed that miR-21 was one of the miRNAs significantly upregulated in HR-HPV (+) and was significantly correlated with radiation resistance in CC. Liu et al. analyzed serum and tissue samples from 22 patients with CC and 20 healthy individuals, and showed that miR-21 was highly expressed in serum and tumor tissues of patients with HR-HPV(+) CC. In addition, analysis of serum and tissue samples from patients with CC 6 months after radiotherapy showed that miR-21 expression was higher in the radiotherapy-resistant group than in the radiotherapy-sensitive group. Meanwhile, the expression trend of large tumor suppressor kinase 1 (LATS1) was opposite to that of miR-21. *In vitro* studies have shown that miR-21 can reduce the radiosensitivity of HR-HPV (+) CC cells, thereby reducing G2/M block and increasing the number of S-phase cells. By targeting the binding site between miR-21 and the LATS1 3’-UTR, miR-21 regulates LATS1 expression in CC cells through direct binding, and overexpression of LATS1 can reverse the colony formation rate induced by miR-21, as well as reduce the accumulation of S phase and G2/M phase arrest induced by miR-21. These studies suggest that miR21-LATS1 is closely related to the radiosensitivity of CC (181). In addition, miR-181a and miR-106b were highly expressed in CC tissues or CC cell lines, and such high expression levels were related to the resistance to radiotherapy in CC (180, 182).

With the development of medical science and technology and detection equipment, miRNAs, as a new molecular marker, have broad application prospects in the diagnosis and treatment of malignant tumors. In CC, miRNAs are mainly involved in the study of cisplatin and radiation resistance, but the clear mechanism of action remains unclear. However, with the deepening of miRNA research, the mechanism by which miRNAs regulate cisplatin or radiation resistance may be clarified. In the future, targeted miRNAs may be used as CC chemotherapy/radiation sensitization agents.

### 3.8 Resistance to radiation therapy for cervical cancer and lncRNAs

LncRNAs, a group of nonprotein coding RNAs with a length of more than 200 nucleotides, regulate a variety of biological processes in a variety of malignant tumors (183–186). In recent years, many studies have shown that lncRNAs are correlated with the radiosensitivity of malignant tumors (187, 188). Recently, lncRNAs have also been reported to play a positive role in CC radiotherapy resistance.

Recent studies have shown that lncRNA GAS5 is closely related to radiotherapy sensitivity in CC. Gao et al. (182) detected GAS5 in 9 radioresistant and 11 radiosensitive CC tissues and found that GAS5 expression in radioresistant tissues was significantly reduced compared with that in radiosensitive tissues. *In vitro* studies showed that GAS5 overexpression could reduce the cell survival fraction and promote radiotherapy sensitivity, while GAS5 knockdown could increase the cell survival fraction and lead to radiotherapy resistance. Animal experiments also confirmed that the tumor size of the GAS5 overexpression group was smaller, while that of the GAS5 knockdown group was larger. Further mechanistic studies showed that GAS5 enhanced the sensitivity of CC cells to radiotherapy by inhibiting miR-106b to upregulate IER3. The above research results indicate that low expression of lncRNA GAS5 can lead to radiotherapy resistance the CC. However, there are few clinical reports on these lncRNAs, and most lncRNAs have increased expression in CC. Therefore, the low expression of LncRNA GAS5 in CC is still worthy of further study. Studies have shown that HOTAIR is highly expressed in CC tissues and cells, and is associated with tumor proliferation and metastasis (189, 190). In addition, HOTAIR regulates the sensitivity of CC to radiation therapy. When Li et al. (191) studied the sensitivity of HOTAIR to radiotherapy for CC, they found that the increased expression of HOTAIR led to the resistance of C33A cells to radiation, causing tumor cells to enter the S phase, but this effect could be reversed by the overexpression of p21, and the downregulation of HOTAIR expression significantly increased the radiation sensitivity. The results suggest that HOTAIR regulates the sensitivity of CC cells in relation to p21. The same study also showed that the expression of HOTAIR in the normal cervix was significantly lower than that of in HeLa and C33A cells. After irradiation, the activity of CC cells was inhibited and the apoptosis of CC cells was promoted. However, this effect could be eliminated by overexpression of HOTAIR. Further mechanistic studies indicated that HOTAIR regulates radiotherapy sensitivity and HIF-1α expression in CC cells. In HeLa and C33A cells with high HOTAIR expression, HIF-1α expression can be increased after radiotherapy, leading to radiation resistance and tumor growth, but this effect can be neutralized by miR-217 mimics. Considering that hypoxia can upregulate HOTAIR’s inhibition of miR-217 expression, the expression of HIF-1α is promoted to increase the radiation resistance of CC cells (192). In addition, LncRNAs associated with radiosensitivity of cervical cancer include UCA1, LINC00662, LINC00958, LINP1, MALAT1, NEAT1, PCAT1 and SNHGs (132, 193–200).

LncRNAs regulate the radiosensitivity of CC mainly by mediating DNA damage, inhibiting tumor cell growth, regulating the cell cycle and apoptosis, and glycolysis and targeting miRNAs. Therefore, the regulation of radiation resistance in CC by lncRNAs has become one of the important directions of current research on the radiation sensitivity of malignant tumors. However, in clinical treatment implementation, there are no clear guidelines based on any lncRNA expression levels to distinguish patients with CC who are sensitive to radiation therapy from those who are resistant to radiation therapy. However, there are no guidelines for using lncRNA expression levels in clinical diagnosis and treatment to distinguish CC patients who are sensitive to radiation therapy from those who are resistant to radiation therapy. However, with the development of detection technology and analysis technology, we can predict the efficacy and prognosis of patients with malignant tumors by monitoring the expression level of lncRNA and related signaling pathways, so that patients with malignant tumors can obtain accurate treatment and individualized treatment, and improve the effect of radiotherapy for patients with CC.

## 4 Conclusion and outlook

Radiotherapy is an important treatment method for CC, but resistance to radiotherapy has become the main obstacle in the treatment of CC patients. At present, there have been many studies and reports on the mechanism of radiation resistance in CC. Most findings support that radiation therapy resistance is associated with a reduction in CC cell death due to enhanced DNA repair ability after radiation therapy. In addition, glucose metabolism is also a very important factor in the occurrence of CC radiation resistance. Lactic acid produced by glycolysis may promote the growth and proliferation of CC cells and may also promote the radiation resistance of CC cells. Therefore, the intermediate products of the glycolysis process are expected to become a new product to improve radiation therapy resistance. Malignant stem cells are naturally resistant to drugs, including radiation therapy. Their survival and adaptability are factors that regulate their maintenance, thus affecting tumor treatment efficacy and leading to tumor recurrence or metastasis. Therefore, it is necessary to fully understand the specificity and nature of tumor stem cells and develop new therapeutic targets to improve the therapeutic efficacy of radiotherapy, targeting, and immunity. In addition, radiotherapy resistance in CC can also be induced by hypoxia, the tumor microenvironment, amino acid metabolism, lipid metabolism, the cell cycle and apoptosis, and miRNAs and lncRNAs after radiotherapy, thus promoting tumor recurrence and metastasis. However, the mechanism of CC radiation therapy resistance is not influenced by a single mechanism, but may be the result of multiple mechanisms. For example, in the tumor microenvironment, there may be multiple mechanisms such as hypoxia, glycolysis, and CSCs that promote radiation therapy resistance.

Here, we summarize the current reported resistance mechanisms of CC in radiotherapy treatment. However, the mechanism of resistance in radiotherapy treatment is complex and diverse, and there may still be unknown mechanisms of action, which still need to be further explored and discovered. In addition, we also discuss how to overcome radiotherapy resistance and improve the curative effect of radiotherapy for CC. It is an important challenge for clinical workers and researchers to improve the sensitivity of radiotherapy for CC by targeting relevant targets or pathways. Similarly, how to select early and sensitive biomarkers to predict, prevent, or control radiation therapy resistance is an important topic for us to study. In addition, we need to strengthen research on radiophysics and radiobiology, so as to provide a theoretical basis for the inhibition of radiation therapy resistance and carry out clinical translational applications.

## Author contributions

HZ and XW designed the research. HZ drafted the manuscript. HZ, YM, RL, and HL improved the structure of this manuscript and completed the diagrams. XW, QZ, and ZW discussed and revised the manuscript. XHW and ZW put forward some constructive suggestions. All authors read and approved the final manuscript.

## Glossary

**Table:**

CC	cervical cancer
RT	radiotherapy
HPV	human papillomavirus
STI	sexually transmitted infection
LACC	locally advanced cervical cancer
ROS	reactive oxygen species
RR	radiotherapy resistance
MDSC	medullogenic suppressor cells
ATM	ataxic telangiectasia mutation
HR	homologous recombination
NHEJ	nonhomologous end-joining
HIF	HYPOXIC inducible factor
RFS	recurrent-free survival rate
DDR	DNA damage response
ECM	extracellular matrix
TME	tumor microenvironment
CSC	cancer stem cell
GLUT1	glucose transporter 1
PPP	pentose phosphate pathway
PK	pyruvate kinase
HK2	hexokinase 2
COX	cyclooxygenase
GLS2	glutaminase-2
OCT4	octamer binding transcription factor 4
SOX2	sry-related HMG box
Sp1	specific protein 1
LAST1	large tumor suppressor kinase 1
LncRNA	long non-coding RNA
GAS5	growth arrest special 5
HOTAIR	HOX transcript antisense intergenic RNA
LINC00662	long intergenic noncoding RNA00662
LINC00958	long intergenic noncoding RNA00958
LINP1	LncRNA in non-homologous end joining (NHEJ) Pathway 1
MALAT1	metastasis-associated lung adenocarcinoma transcript 1
NEAT1	nuclear-enriched transcript 1
PCAT1	prostate cancer-associated transcript 1
SNHG	small nucleolar RNA host gene
UCA1	urothelial cancer associated 1
